# Activated MC_TC _mast cells infiltrate diseased lung areas in cystic fibrosis and idiopathic pulmonary fibrosis

**DOI:** 10.1186/1465-9921-12-139

**Published:** 2011-10-20

**Authors:** Cecilia K Andersson, Annika Andersson-Sjöland, Michiko Mori, Oskar Hallgren, Annie Pardo, Leif Eriksson, Leif Bjermer, Claes-Göran Löfdahl, Moises Selman, Gunilla Westergren-Thorsson, Jonas S Erjefält

**Affiliations:** 1 Dept of Respiratory Medicine and Allergology, Lund University, Klinikgatan 30, 221 84 Lund, Sweden; 2 Dept of Exp Medical Science, Lund University, Klinikgatan 30, 221 84 Lund, Sweden; 3 Faculty of Sciences, Universidad Nacional Autónoma de México, Avenida Universidad 3000; CP 04510 Mexico DF, México; 4 Instituto Nacional de Enfermedades Respiratorias "Ismael Cosío Villegas", Tlalpan 4502; CP 14080, México DF, México

**Keywords:** mast cell, connective tissue mast cell, cystic fibrosis, idiopathic pulmonary fibrosis, alveolar parenchyma, remodelling, fibrosis

## Abstract

**Background:**

Although mast cells are regarded as important regulators of inflammation and tissue remodelling, their role in cystic fibrosis (CF) and idiopathic pulmonary fibrosis (IPF) has remained less studied. This study investigates the densities and phenotypes of mast cell populations in multiple lung compartments from patients with CF, IPF and never smoking controls.

**Methods:**

Small airways, pulmonary vessels, and lung parenchyma were subjected to detailed immunohistochemical analyses using lungs from patients with CF (20 lung regions; 5 patients), IPF (21 regions; 7 patients) and controls (16 regions; 8 subjects). In each compartment the densities and distribution of MC_T _and MC_TC _mast cell populations were studied as well as the mast cell expression of IL-6 and TGF-β.

**Results:**

In the alveolar parenchyma in lungs from patients with CF, MC_TC _numbers increased in areas showing cellular inflammation or fibrosis compared to controls. Apart from an altered balance between MC_TC _and MC_T _cells, mast cell in CF lungs showed elevated expression of IL-6. In CF, a decrease in total mast cell numbers was observed in small airways and pulmonary vessels. In patients with IPF, a significantly elevated MC_TC _density was present in fibrotic areas of the alveolar parenchyma with increased mast cell expression of TGF-β. The total mast cell density was unchanged in small airways and decreased in pulmonary vessels in IPF. Both the density, as well as the percentage, of MC_TC _correlated positively with the degree of fibrosis. The increased density of MC_TC_, as well as MC_TC _expression of TGF-β, correlated negatively with patient lung function.

**Conclusions:**

The present study reveals that altered mast cell populations, with increased numbers of MC_TC _in diseased alveolar parenchyma, represents a significant component of the histopathology in CF and IPF. The mast cell alterations correlated to the degree of tissue remodelling and to lung function parameters. Further investigations of mast cells in these diseases may open for new therapeutic strategies.

## Background

Mast cells release a vast range of cytokines, proteases and proteoglycans that can modify and direct the inflammatory response towards resolution or formation of fibrosis [1]. Mast cell-derived molecules can both modify the production and destruction of extracellular matrix as well as promote migration and proliferation of fibroblasts *in vitro *[2-4]. Despite that mast cells have been shown to promote fibrosis in several organs [5,6], their relationship to fibrotic lesions and inflammatory foci in the lung has remained poorly studied.

The present study involves assessment of mast cells in cystic fibrosis (CF) and idiopathic pulmonary fibrosis (IPF), diseases that have peripheral lung fibrosis as a significant pathological feature. The pathophysiological events in CF are caused by mutations in the chloride channel protein, cystic fibrosis transmembrane conductance regulator (CFTR), leading to defective trans-epithelial ion transport, airway surface liquid depletion, decreased mucociliary clearance and mucus obstruction [7]. Due to these effects decreased clearance of pathogens result in chronic infections, which sustains inflammation and cause lung remodelling and fibrosis [8]. In IPF the pathology is characterised by epithelial damage, mild inflammation, formation of fibroblast foci, and excessive extracellular matrix deposition in the alveolar parenchyma [9,10].

Previous studies have reported presence of mast cells in the airways of patients with CF [11,12] and increased mast cell numbers in the airways of patients with IPF [13-15]. However, information about the distribution pattern and phenotypes of lung mast cells in these conditions is lacking. Mast cells are traditionally divided into two subtypes according to their granule content; tryptase^+ ^and chymase^+ ^mast cells (MC_TC_: "connective tissue mast cells") and tryptase^+ ^but chymase^- ^mast cells (MC_T_: "mucosal mast cells"), where the latter is the most frequent in healthy lungs [16]. Recent data suggest that the proportion of the MC_TC _population is increased in inflammatory airway diseases like asthma and COPD [17-19], and increased chymase expression has been reported in human idiopathic interstitial pneumonia [20,21].

In the present study we hypothesised that the mast cell populations of the lung are significantly altered in CF and IPF, and that this may contribute to the pathogenesis of these disorders. The changes in MC_T _and MC_TC _populations in CF and IPF were studied by histological assessment in key lung compartments and compared to that in never-smoking control subjects. The importance of exploring several anatomical regions is underscored by the discovery that each of the MC_T _and MC_TC _population can be divided into site-specific populations unique for each anatomical compartment [22]. The use of large surgical resections allowed us to compare key compartments such as small airways and pulmonary vessels and alveolar parenchymal regions affected by severe cellular inflammation and/or parenchymal fibrosis. To get further insight into the MC_T _and MC_TC _phenotypes in CF and IPF, mast cell expression of mediators of importance in the inflammatory responses and fibrosis (IL-6 and TGF-β), were analysed in the same lung regions.

## Methods

### Subjects

CF was diagnosed on the basis of clinical manifestations from the lung and gastro-intestinal tract and a positive sweat test [23,24]. In 5 patients with end-stage CF, lung tissues (20 large tissue blocks) were collected in association with lung transplantation. The study also explored 21 large lung tissue blocks from 7 IPF patients, diagnosed based on established criteria [9] and confirmed by open lung biopsy. None of the IPF patients reported any environmental or occupational exposure and the patients were untreated at the time of tissue collection (see Table 1). A group with never-smoking subjects was used as controls (*n *= 8) [17,22]. The control tissue was obtained during lung lobectomy due to suspected lung cancer from otherwise healthy non-atopic individuals [25,26]. Only patients with solid (and typically centrally located) tumours were included and the tissue for the present analysis was collected from peripheral lung regions, as far from the tumour as possible. All CF patients and control subjects gave their written informed consent to participate in the study, which was approved by the ethics committee in Lund, Sweden (FEK 91/2006 and FEK 213/2005). The research protocol for IPF patients, who gave written informed consent to be included in the study, was approved by the Ethics Committee of the National Institute of Respiratory Diseases, Mexico. For patient characteristics, see Table 1.

**Table 1:** Characteristics of patients with CF, IPF and controls

	Controls	CF	IPF
Sex (M/F, n)	2/6, 8	2/3, 5	6/1, 7
Age^a ^(years)	63 (33-76)	30 (23-38)	64 (57-70)
Current smokers (y/n)	0	0	0
Ex-smokers (y/n)	0	0	3/4
Inhaled GCS (y/n)	0	5/0	0/7
Oral GCS (y/n)	0	3/2	0/7
B2 agonist (y/n)	0	5/0	0/7
*Lung function*			
FEV_1 _% of predicted^a^	110 (82-141)	31 (22-45)	82 (66-93)
FEV_1_/(F)VC^a^	86 (66-121)	50 (33-84)	90 (82-99)
VC % of predicted^a^	104 (82-126)	54 (46-70)	66 (42-75)
TLC % of predicted^a^	ND	104 (79-129)	68 (51-90)
RV % of predicted^a^	ND	215 (113-318)	61 (51-101)
% TLCO SB	ND	60 (50-71)	74 (51-110)

^a ^Data are given as mean (range). M = male, F = female, GCS = glucocorticosteroid, FEV1 = forced expiratory volume in 1 second, VC = vital capacity. TLC = total lung capacity. RV = residual volume. TLCO = diffusing capacity for carbon monoxide, ND = not determined.

### Processing of Tissue and Histological Procedures

Lung tissue was dissected and sampled under controlled circumstances in a study that was designed to collect tissue from corresponding areas from each patient. To guarantee optimal antigenicity, care was taken to immerse the tissue in fixative immediately after surgical excision and multiple large tissue blocks were prepared for histological analysis. Lung tissue blocks were placed in 4% buffered formaldehyde, dehydrated, and embedded in paraffin and sequential sections was generated. An initial screening of haematoxylin-stained sections was performed to select tissue blocks encompassing regions of fibrotic lesions, cellular inflammation, and normal tissue areas. One section per tissue block was stained with Masson's trichrome for evaluation of gross pathological changes.

#### Double Immunohistochemical Staining of MC_TC _and MC_T_

**Figure 1:**
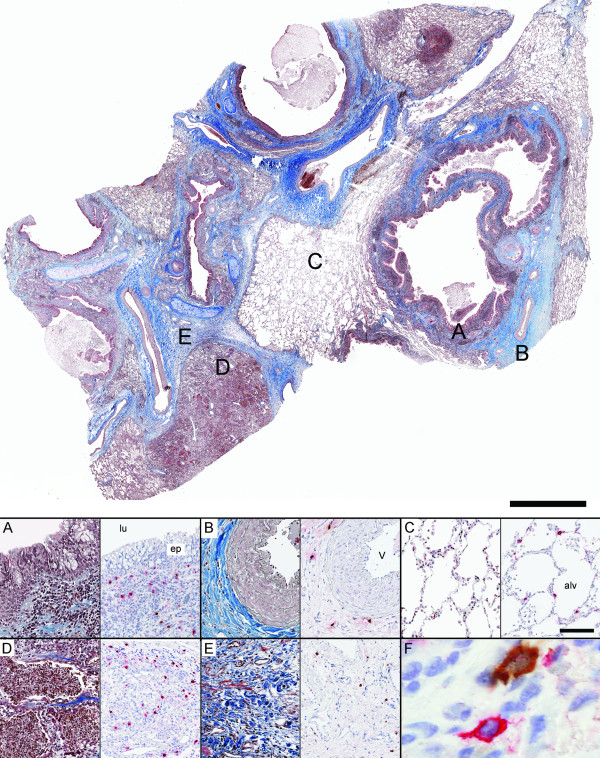
**Representative low magnification overview of a lung section from a patient with CF, stained with Masson's trichrome staining**. Panel A-E show representative micrographs in different anatomical compartments of the lung from a patient with CF: (A) small airway, (B) pulmonary vessel, (C) normal alveolar parenchyma, (D) region of inflammation in alveolar parenchyma and (E) region of fibrosis in alveolar parenchyma. Consecutive sections were used for immunohistochemical double staining of tryptase-positive mast cells (MC_T_: permanent red) and chymase-positive mast cells (MC_TC_: DAB- brown) shown to the right in each panel. F represents a close-up image (600×) of neighbouring MC_TC _and MC_T _cells. Scale bars: Overview = 2000 μm, A-E = 100 μm. lu: airway lumen; ep: small airway epithelium; v: pulmonary vessel; and alv: alveolar parenchyma.

A previously validated double staining protocol was used for simultaneous visualisation of MC_TC _and MC_T _cells (Figure 1F) [17,22]. The staining was performed by an automated immunohistochemistry robot (Autostainer; Dako, Glostrup, Denmark) with EnVision™ G|2 Doublestain System (K5361, Dako)[17]. After rehydration and antigen retrieval, chymase-containing mast cells were detected with an mouse anti-chymase antibody (1:100, Novocastra, Newcastle upon Tyne, UK) a horse-radish peroxidase(HRP)-conjugated anti-mouse secondary antibody and the non-permeable chromogen DAB. A double stain blocking reagent (K5361, Dako) prevented further recognition of anti-chymase antibodies (by chemically destroying the antigenicity of previously applied antibodies) and the remaining MC_T _subclass was visualised with an mouse anti-tryptase antibody (1:12 000, Chemicon, Temecula, CA), an alkaline phosphatase(AP)-conjugated anti-mouse secondary antibody, and Permanent Red chromogen. Sections were stained with Mayer's haematoxylin for visualisation of background.

#### Identification of Mast Cell-Related Molecules using Immunofluorescence

A validated triple immunofluorescence protocol [17,22] was used to simultaneously visualise the mast cell-related molecules interleukin 6 (IL-6) or transforming growth factor beta (TGF-β) together with both MC_TC _and MC_T _subtypes (Table 2). After antigen retrieval and a blocking step with 5% horse serum, the IL-6 (mouse anti-IL-6, 1:50, Novocastra) or TGF-β (mouse anti-TGF-β, 1:40, Novocastra) immunoreactivity was visualised with a horse anti-mouse biotinylated secondary antibody and Alexa-Flour 555-conjugated streptavidin (Molecular Probes, Oregon, USA). Next, MC_T _and the MC_TC _populations were stained with anti-tryptase and anti-chymase antibodies that were pre-labelled with AlexaF-488 fluorochrome and AlexaF-350 fluorochrome, respectively (Zenon IgG1 labelling kit, Molecular Probes).

**Table 2:** Densities and proportions of mast cell populations in different lung compartments

			Controls	CF	p-value	IPF	p-value
Small Airways *(mast cell/mm^2^)*		MC_Tot_	357 (274-530)	89 (59-145)	0.002	357 (199-488)	0.8
		MC_T_	332 (204-500)	65 (42-121)	0.002	197 (62-267)	0.004
		MC_TC_	23 (17-73)	23 (9-74)	0.7	156 (114-249)	0.0003
		%MC_TC_	6 (4-26)	30 (13-54)	0.01	52 (42-69)	0.0003
Pulmonary Vessels *(mast cell/mm^2^)*		MC_Tot_	217 (124-522)	30 (24-60)	0.002	132 (89-260)	0.03
		MC_T_	123 (38-338)	15 (6-34)	0.002	21 (9-42)	0.0006
		MC_TC_	89 (28-185)	18 (14-26	0.002	97 (77-223)	0.05
		%MC_TC_	46 (13-70)	59 (47-83)	0.2	84 (71-93)	0.0003
Alveolar parenchyma *(mast cell/mm^2^)*	Normal	MC_Tot_	28 (9-85)	58 (39-151)	0.1	65 (15-120)	0.1
		MC_T_	26 (9-83)	52 (32-61)	0.2	54 (12-107)	0.2
		MC_TC_	2 (0-6)	6 (3-90)	0.02	12 (2-16)	0.01
		%MC_TC_	5 (0-11)	18 (5-60)	0.03	17 (11-18)	0.005
	Inflam-matory	MC_Tot_	-	96 (59-150)	0.02*	193 (74-274)	0.0006*
		MC_T_	-	73 (53-115)	0.02*	88 (15-114)	0.05*
		MC_TC_	-	7 (5-65)	0.01*	105 (59-171)	0.001*
		%MC_TC_	-	14 (4-42)	0.07*	62 (43-80)	0.001*
	Fibrotic	MC_Tot_	-	87 (77-137)	0.006*	192 (64-327)	0.001*
		MC_T_	-	70 (44-78)	0.07*	105 (11-181)	0.009*
		MC_TC_	-	20 (10-69)	0.004*	88 (19-147)	0.001*
		%MC_TC_	-	22 (16-51)	0.004*	49 (24-82)	0.001*

Data presented as median (range). p-value is considered significant ≤ 0.05. *Denotes comparison to normal parenchyma in control subjects.

#### Antibody Specificity Controls

All antibodies in the present studies have been used extensively in research and routine pathological examinations of human formalin-fixed, paraffin-embedded material; most antibodies have also been validated and certified for pathology-based diagnosis. For all immunohistochemical procedures, markers and tissues, staining was absent in sections using isotype-matched control antibodies (Dako) that were used instead of, and in the same concentration as, the primary antibody. The mouse monoclonal isotype IgG1 and 2 controls are antibodies against Aspergillus niger glucose oxidase, an enzyme which is neither present nor inducible in mammalian tissues. The rabbit negative control immunoglobulin fraction was isolated from serum from healthy non-immunized rabbits.

### Measurements

#### Quantification of Mast Cell Densities in Major Lung Compartments

From each section double stained for MC_T _and MC_TC_, an ultra-high resolution digital image (> 4 GB) was generated by an automated slide scanner robot equipped with a x20 microscope lens (ScanScope FL, Aperio, Vista, CA). Using virtual microscopy software (Image Scope, v10.0.36.1805, Aperio) the density of each population was quantified manually in blinded images (CF: 20 lung blocks, 4 per patient; IPF: 21 lung blocks, 3 per patient; controls: 16 blocks, 2 per patient) and related to the tissue area. After manual delineation of the each analysed compartment, the analysed tissue area was calculated through computerised image analysis (ImageScope, Aperio, Vista, CA). Each tissue section comprised > 4 cm^2 ^lung tissue and contained several small airway walls, small airway lumen, pulmonary vessel walls as well as differentially altered alveolar parenchyma, allowing for comparisons between the following key compartments; *Small airways: *bronchioles, defined by absence of cartilage and diameter < 2 mm. *Pulmonary vessels: *mid-size pulmonary arteries in the broncho/bronchiovascular axis or with an intra-acinar localisation. The number of vessels per section was related to the tissue area and a mean number of vessels/mm^2 ^per patient were calculated. *Alveolar parenchyma*: Due to the heterogeneity of the alveolar parenchyma in CF and IPF, each slide was assessed for mast cells in the following category of parenchyma: normal, or nearly normal parenchyma (defined as absent or minimal fibrous thickening of alveolar walls and only few scattered inflammatory cells); inflammatory parenchyma (areas with significant leukocyte infiltration, often accompanied by some alteration of alveolar structure); and fibrotic parenchyma (fibrotic lesions and regions with excessive collagen deposition, as revealed by Masson's trichrome staining in consecutive sections). Four randomly selected 0.5 mm^2 ^alveolar regions were analysed in each lung section for each category of parenchyma. The proportion (%) of the MC_TC _subtype in each compartment was calculated according to (MC_TC_/[MC_TC _+ MC_T_]) ×100.

#### Expression of Mast Cell-Related Molecules, IL-6 and TGF-β

The markers were selected to represent possible roles for mast cells in chronic inflammation (IL-6) and in the formation of fibrosis (TGF-β). Sections were analysed using NIS-elements AR 3.0 system (Nikon, Tokyo, Japan), a Nikon Eclipse 80i microscope, and a Nikon DS-Qi1Mc camera. In triple stained sections, all tryptase- and chymase-positive cells in the walls of 4 randomly selected small airways, 4 pulmonary vessel walls, and 4 randomly selected 0.5-mm^2 ^alveolar regions per lung section were analysed for IL-6 and TGF-β. By subsequently dividing the number of tryptase and/or chymase cells co-positive for IL-6, TGF-β, respectively, by the total numbers of MC_T _and MC_TC_, the proportion (%) of each subtype and the total number of mast cells expressing each mast cell-related molecule were obtained.

#### Measurements of degree of fibrosis

The degree of fibrosis was analysed in randomly selected 0.5 mm^2 ^alveolar regions stained with Masson's trichrome, using a scoring system for fibrosis [27]. On the same slides, tissue density (per um^2^) of collagen was assessed by computerised image analysis using ImageScope (Aperio, Vista, CA).

### Statistical Analysis

Data were analysed statistically using Mann-Whitney rank sum test for comparison between two groups using GraphPad Prism v. 5 (GraphPad Software Inc., La Jolla, CA). Data were analysed statistically using Kruskal Wallis test with Bonferroni's multiple comparisons test for comparison between three groups or more. Mast cell parameters were correlated (Spearman rank correlation test two-tailed) to lung function and degree of fibrosis for CF and IPF patients. Due to the low numbers of patients, correlation analyses were also performed on pooled CF and IPF patients. The mean number of vessels per patient was correlated to mast cell parameters within the IPF group. For all outcomes, a p-value ≤ 0.05 was considered significant (* denotes p ≤ 0.05, ** p < 0.01 and *** < 0.001).

## Results

### General histopathological description

**Figure 2:**
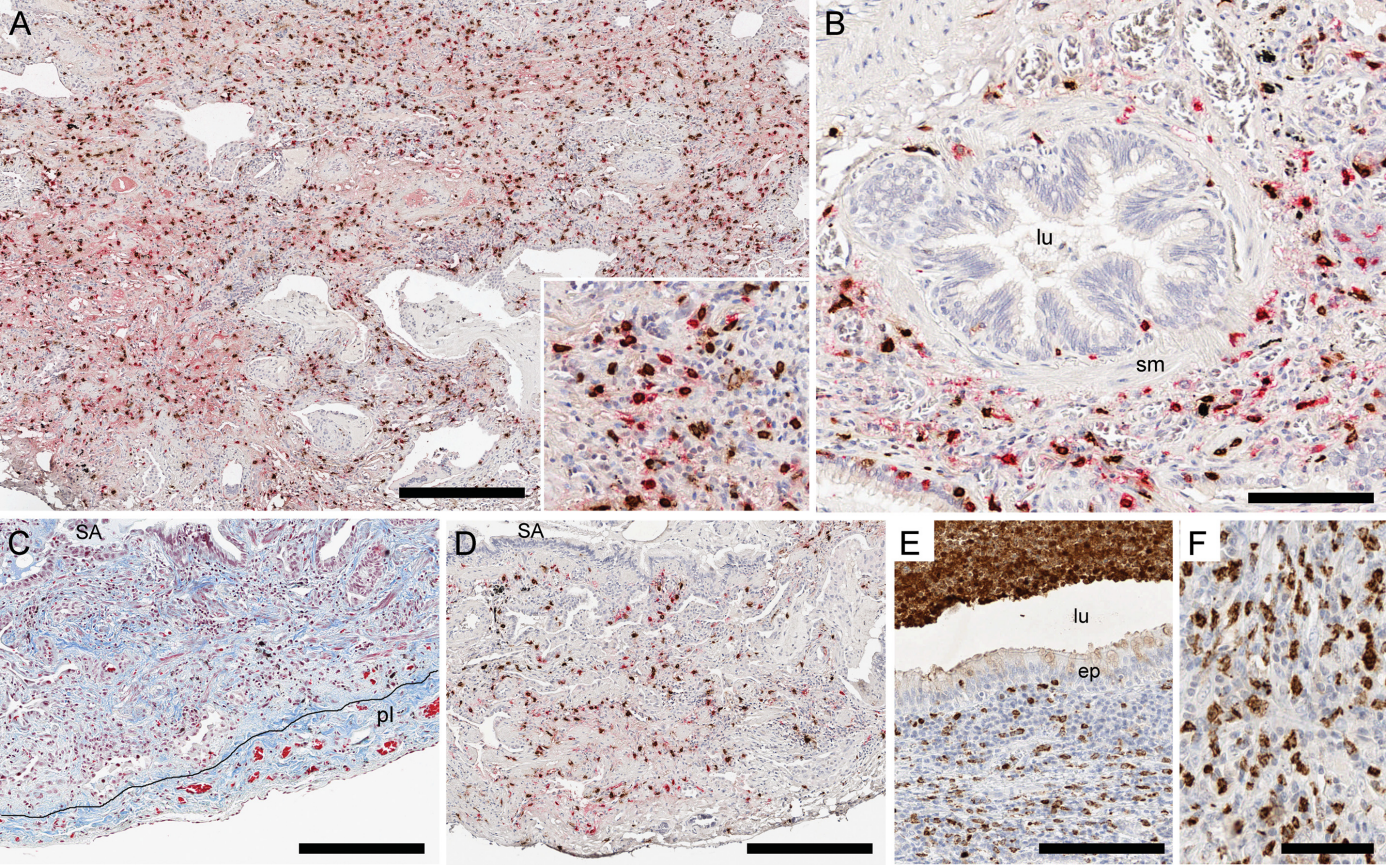
**Representative micrographs from a patient with IPF**. Panels A-B show lung section stained with immunohistochemical double staining for tryptase-positive mast cells (MC_T_: permanent red) and chymase-positive mast cells (MC_TC_: DAB- brown): alveolar parenchyma with interstitial fibrosis (A) and small airway (B). Picture C shows alveolar parenchyma with interstitial fibrosis and pulmonary pleura. High density of collagen is seen in blue with trichrome staining and picture D shows the corresponding area from a consecutive section double stained for MC_T _and MC_TC_. In picture E lung tissue from a patient with CF is shown. High density of MPO^+ ^neutrophils is seen in the subepithelial tissue, in the airway lumen (E), and in the alveolar parenchyma (F). Scale bars: A, C-D = 300 μm, B and E = 100 μm and F = 50 μm. lu: airway lumen; ep: small airway epithelium; v: pulmonary vessel; pl: pleura and alv: alveolar parenchyma.

Sections stained with Masson's trichrome revealed extensive alterations of lung structures in all CF and IPF patients (Figure 1 and 2). In CF, changes of the small airways included metaplastic and damaged epithelium, subepithelial inflammation and mucopurulent plugging (Figure 1A). The alveolar compartment was affected by structural changes, although patchy areas of normal, or nearly normal, tissue were also present (Figure 1C). Pneumonia was common, with infiltration of inflammatory cells in the alveolar walls, intra-alveolar exudate (alveoli filled with mainly neutrophils, Figure 1D) and presence of lymphoid aggregates. Fibrotic regions were present in the adventitia of small airways (Figure 1A) and pulmonary vessels (Figure 1B) and in the alveolar interstitium (Figure 1E). The pathology of the IPF patients (Figure 2) was characterised by deformation of normal lung architecture including honeycombing and formation of fibrotic and fibroblast foci. The alveolar parenchyma had widespread interstitial fibrosis, areas of infiltration of inflammatory cells (mostly lymphocytes and mononuclear cells) as well as patchy regions of structurally normal parenchyma.

### Change in Densities of MC_T _and MC_TC _populations in key anatomical compartments in CF and IPF compared to controls

#### Cystic Fibrosis

**Figure 3:**
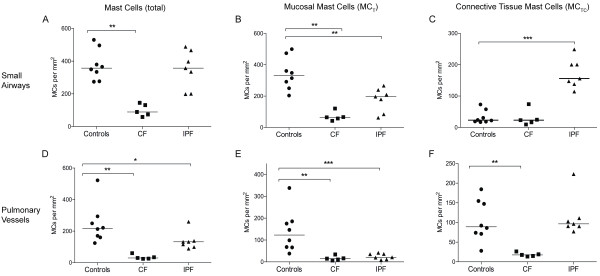
**Total mast cell densities (A, D) and densities of each subtype, MC_T _(B, E) and MC_TC _(C, F), in lung tissue compartments of never-smoking controls and patients with CF and IPF**. Data are presented as mast cells per mm^2 ^lung tissue and presented for small airways (A-C) and pulmonary vessels (D-F). Horizontal line denotes median value. Data are expressed as scatter plots where horizontal lines denote median values. Statistical differences to never-smoking controls using Mann-Whitney where * denotes p < 0.05, and ** p < 0.01.

**Figure 4:**
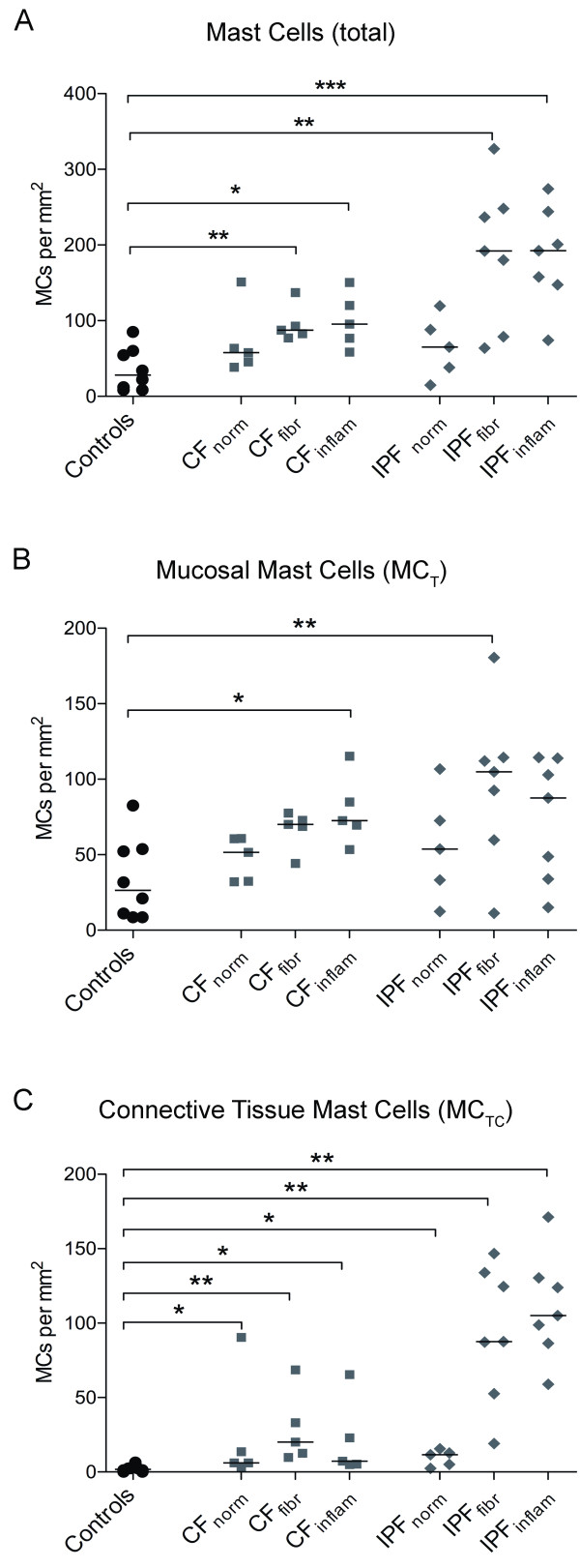
**Mast cell densities in alveolar parenchyma from never-smoking controls and patients with CF and IPF**. Data are presented as mast cells per mm^2 ^lung tissue. The total mast cell densities (A) and densities of each subtype, MC_T _(B) and MC_TC _(C), are presented in regions of normal, inflammatory and fibrotic alveolar parenchyma in each disease. Data are expressed as scatter plots where horizontal lines denote median values. Statistical differences to never-smoking controls using Mann-Whitney where * denotes p < 0.05, ** < 0.01, and *** p < 0.001.

In patients with CF, a significant decrease in total mast cell density was observed in small airways. This reduction was due to decreased MC_T _numbers whereas the density of MC_TC _was unchanged (Figure 3A-C and Table 2). A decrease in total mast cell numbers was observed in pulmonary vessels. In this compartment, the density of MC_T _and MC_TC _decreased (Figure 3D-F and Table 2). In normal areas of the parenchyma in CF affected lungs, the total mast cell number as well as the density of MC_T _was unchanged (Figure 4A-C and Table 2), however, an increase in MC_TC _numbers was observed. The total number of mast cells was increased in fibrotic and inflammatory areas of the alveolar parenchyma in CF patients (Figure 4A and Table 2). In the inflammatory parenchyma this was due to increased numbers of MC_T _and MC_TC _whereas only the density of the MC_TC _population increased in fibrotic parenchyma (Figure 4B-C and Table 2). An increase in total mast cell numbers and the density of MC_T _were found in the small airway lumen in CF patients (MC_tot_: 1 0 [1-15] MC/mm^2^, and MC_T_: 0 0 [1-15] MC/mm^2^) compared to controls (MC_tot_: 0 [0-1.7] MC/mm^2^, p = 0.2 and MC_T_: 0 0 [1,2] MC/mm^2^, p = 0.2). No difference in the density of MC_TC _was found between CF patients and control subjects.

#### Idiopathic Pulmonary Fibrosis

In small airways of IPF patients, no change in the total mast cell density was observed (Figure 2B and 3A). The preserved total mast cell numbers were a result of a decrease in MC_T _density and a parallel increase in MC_TC _density (Figure 3A-C). A significant decrease in total mast cell density due to decreased MC_T _and unchanged MC_TC _was observed in pulmonary vessels (Figure 3D-F and Table 2). In normal areas of IPF lungs, total mast cell numbers as well as the density of MC_T _was unchanged while the density of MC_TC _was increased (Figure 4A-C and Table 2). In fibrotic parenchyma, a significant increase in the density of the MC_T _and MC_TC _population was detected (Figure 2A, C-D). An increase in the density of MC_TC _population was found in the inflammatory parenchyma of IPF patients, but the number of MC_T _was unchanged (Figure 4B-C and Table 2).

### Relative proportion of MC_TC_

**Figure 5:**
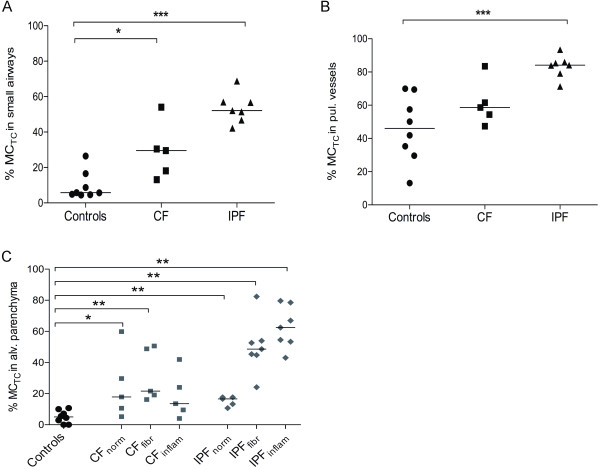
**The proportion of MC_TC _and MC_T_, expressed as the percentage of MC_TC_, in anatomical lung compartments in never-smoking controls and patients with CF and IPF**. Data are presented for small airways (A), pulmonary vessels (B), and alveolar parenchyma (C). Data are presented as scatter plots, where horizontal lines denote median values. Statistical differences to never-smoking controls were analysed using Mann-Whitney and asterisks show significant difference where * denotes p < 0.05, ** < 0.01, and *** p < 0.001.

Calculation of the MC_TC _percentage of the total mast cell population revealed that there was a shift towards a MC_TC _phenotype in lungs from patients with CF and IPF (Figure 5A-C). In CF, the increase in the proportion of MC_TC _cells was found in small airways, and in normal as well as fibrotic areas of parenchyma. In IPF affected lungs, an increase in the proportion of MC_TC _was found in small airways, pulmonary vessels as well as in normal, inflammatory and fibrotic areas of the parenchyma (Figure 5, Figure 2A-D and Table 2).

### Increased mast cell expression of IL-6 and TGF-β in CF and IPF lungs

**Figure 6:**
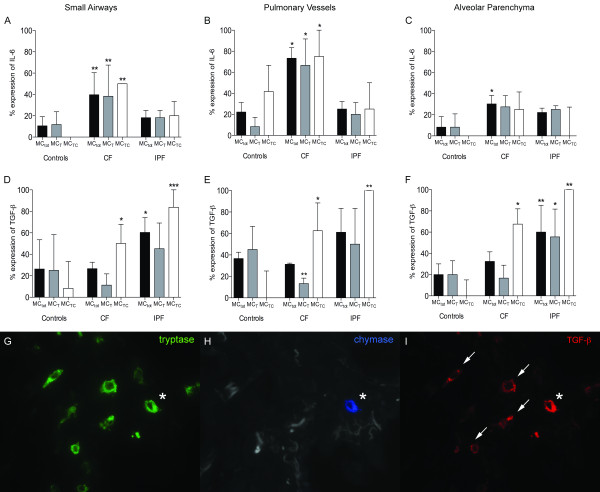
**Expression patterns of interleukin (IL)-6 (A-C), and TGF-β (D-F) in small airways, pulmonary vessels, and alveolar parenchyma**. In each graph, results are shown as the percentage of total mast cells, and of MC_TC _and MC_T _subtypes that are positive for the respective mediator. Data are expressed as medians with interquartile ranges. Statistical differences to never-smoking controls were analysed using Mann-Whitney and asterisks show significant difference where * denotes p < 0.05, ** < 0.01, and *** p < 0.001. Representative micrographs of triple staining of mast cell subtypes and TGF-β (G-I). Arrows indicate tryptase and TGF-β co-localisation and * indicates co-localisation of tryptase, chymase and TGF-β.

The results of IL-6 and TGF-β expression are shown in Figure 6. In CF patients, the total mast cell (MC_tot_) expression of IL-6 was increased in small airways (40 [25-75]%), pulmonary vessels (73 [48-83]%) and alveolar parenchyma (30 [20-39]%) compared to controls (small airways: 11 [0-22]%, p = 0.006; pulmonary vessels: 22 [0-73]%, p = 0.01; and alveolar parenchyma: 8 [0-56]%, p = 0.05). No difference in mast cell expression of IL-6 was observed in the IPF lungs compared to control subjects.

A significantly increased MC_TC _expression of TGF-β was observed in all compartments analysed: IPF (small airways: 84 [75-100]%, pulmonary vessels: 100 [83-100]% and alveolar parenchyma: 100 [50-100]%) and controls (small airways: 8 [0-50]%, p = 0.0008; pulmonary vessels: 0 [0-50]%, p = 0.005; and alveolar parenchyma: 0 [0-20]%, p = 0.004). A similar increase in TGF-β expression was observed in the MC_TC _population in CF patients.

**Figure 7:**
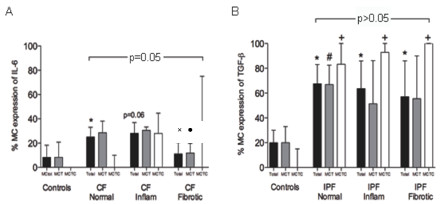
**Mast cell expression of IL-6 in parenchyma in patients with CF (A), and mast cell expression of TGF-β in parenchyma in IPF patients (B)**. Overall significance for total mast cell expression in different parenchyma is shown in each picture. Significance to healthy controls is shown as: p < 0.05 for total mast cell expression (*), as well as MC_T _(#) and MC_TC _(+), and to normal CF parenchyma as: p < 0.05 for total mast cell expression (×) and MC_T _(●).

When dividing the alveolar parenchyma of CF and IPF patients in normal, inflammatory and fibrotic regions, no difference in mast cell expression of TGF-β was found between the different types of parenchyma. The mast cell expression of IL-6 was high in seemingly normal and inflammatory regions of the parenchyma. However, IL-6 expression in mast cells was significantly lower in fibrotic parenchyma compared to normal and inflammatory regions of the CF lung parenchyma (Figure 7).

### Correlation of mast cell parameters to lung function and degree of fibrosis

Some mast cell parameters correlated significantly with lung function values of the CF and IPF patients. As an example, the percentage of MC_TC _in small airways correlated negatively with FEV_1_/VC. Also the density of MC_TC _per mm^2 ^in fibrotic alveolar parenchyma correlated negatively with FEV_1 _% predicted (p_s _= -0.83, p = 0.003). The mast cell expression of TGF-β in different compartment correlated negatively with several lung function values. There were negative correlation between the expression of TGF-β in small airways and FEV_1_/VC (p_s _= -0.90, p = 0.005) as well as between expression of TGF-β in pulmonary vessels and FEV_1 _% predicted (p_s _= -0.86, p = 0.01). Within the IPF group, FEV_1_/VC and the proportion of MC_T _in pulmonary vessel walls correlated negatively (r_s _= -0.87, p = 0.03). There was also a tendency for a negative correlation between FEV_1 _and the proportion of MC_TC _in fibrotic parenchyma (r_s _= -0.83, p = 0.06). In alveolar parenchyma in CF and IPF patients, the density as well as the percentage of MC_TC _correlated positively with the degree of fibrosis (p_s _= 0.73, p < 0.0001 and p_s _= 0.66, p < 0.0001, respectively) and the density of collagen (p_s _= 0.72, p < 0.0001 and p_s _= 0.68, p < 0.0001, respectively). Since mast cells may contribute to vascular remodelling and angiogenesis, mast cell parameters were correlated to the number of vessels per patient. In IPF patients, the number of vessels/mm^2 ^and the density of MC_TC_/mm^2 ^in fibrotic parenchyma showed a correlation of r_s _= -0,75 and p = 0,06.

## Discussion

The present study provides further insight into the mast cell involvement in CF and IPF by providing a detailed characterisation of how each of the major mast cell populations are distributed in the major lung compartments and how they relate to distinct pathologies, including fibrotic lesions. Apart from changes in densities, distribution, and expansion of MC_TC_, both MC_T _and MC_TC _populations displayed an altered expression of IL-6 and TGF-β. Moreover, we show correlations between the increased density of MC_TC _and degree of fibrosis in CF and IPF patients.

Although the present study produced clear and conclusive results, the study has some limitations that need to be discussed. All control patients in the present study were undergoing lobectomy for suspected lung cancer. Theoretically the presence of a tumour might have an effect on mast cell properties. To partly overcome this problem we analysed only lung regions collected far from the tumour, a procedure has commonly been used to collect human control tissue [25,28]. Furthermore, we compared the control tissue in the present study with a separate material of bronchial and transbronchial biopsies from completely healthy subjects and have found no differences in mast cell parameters [22,29]. Due to difficulties in obtaining the present type of immediately fixed lung tissue, the number of patients in the CF group was relatively low. Yet, many of the mast cell parameters were markedly and statistically altered in IPF and CF compared to controls. The lack of statistical differences between the study groups, however, must be interpreted with cautiousness (although we have performed a power study for the non-significant results that showed that for most parameters the power is sufficient to rule-out any clear alterations in the absence of statistical differences). To partly compensate for the limited patient numbers in the present study, the strategy was indeed to, for each patient, perform a detailed characterisation of mast cells in multiple lung regions and, in addition, in each tissue block also study numerous sub-compartments subjected to different types of pathologies.

Our demonstration of a shift in the balance between the mast cell subtypes in the analysed compartments, with a several-fold increase in the percentage of the MC_TC _subtype in fibrotic lesions represents a key finding in the present study. Similar expansion of the MC_TC _population has also been observed in other respiratory diseases like asthma [18] and COPD [17]. The expansion of the MC_TC _population in CF and IPF may have numerous implications in lung remodelling processes. In support of this, Hirata *et al*. [20] showed increased chymase expression in human idiopathic interstitial pneumonia, correlated with increased numbers of IL-4 expression cells, smooth muscle cells and myofibroblasts. Furthermore, Tchougounova *et al*. [30] demonstrated a possible role for chymase in mMCP-4 knockout mice, where chymase-deficient mice developed an imbalance in extracellular matrix production. Increased chymase expression and mast cell involvement have also been demonstrated lung vascular remodelling in pulmonary hypertension [31,32]. In IPF patients, we found a tendency for a negative correlation between the number of vessels/mm^2 ^and the density of MC_TC_.

Increased numbers of mast cells have been found in animal models of fibrosis [33,34] and mast cells have been shown to affect biochemical properties of lung fibroblast function *in vitro *[2,4,35]. Increased numbers of mast cells in patients with fibrotic lung disorders have been described by others [14,21] and some studies described a association to the degree of fibrosis [15]. Hubeau *et al*[12] found unaltered mast cell numbers in the conducting airways in CF lungs. Unfortunately, other compartments of the lung were not included in this investigation. Little is currently known regarding the recruitment mechanisms of mast cells to diseased areas of the lung [36]. Mast cell progenitor recruitment to the airways is dependent on integrins and mediated by several chemotactic factors (chemokines, leukotrienes etc.) [37-39]. For example, mast cells have been shown to adhere to airway smooth muscle via cell adhesion molecule-1 (CADM1) through activation of the CXCR3/CXCL10 axis [40,41]. This is an important filed of research for completely elucidating the role of mast cells in disease.

Due to the differences in pathogenesis in CF and IPF, we have avoided making any direct comparisons of the two diseases. Although CF and IPF have different etiologies, the basic pathological features of the fibrotic lesions include excessive collagen deposition. Our data show that mast cells infiltrate these regions. We showed strong statistical correlations between increased densities of MC_TC _cells, degree of fibrosis and parenchymal collagen density. These observations points toward a role for mast cells in the pathogenesis of fibrotic lesions and is further supported by our observation that expression of TGF-β, was increased in the MC_TC _population in CF and IPF lungs. The importance of TGF-β as a fibrotic agent in the lung has been widely demonstrated [42,43]. An increased expression of TGF-β has been documented during tissue remodelling in several chronic lung diseases including CF [44] and in IPF [45]. In mice and rat lungs a severe fibrotic response develops in the lung after induction of active TGF-β [46,47]. Injuries of human lung tissue induced by different stimuli including bacteria lead to an induction of TGF-β, which plays a key role in mediating tissue remodelling and repair. TGF-β, when expressed in the airways stimulates fibroblasts to produce extracellular matrix proteins [43,48].

Mast cells in CF and IPF lungs may also participate in inflammatory events. Although lung tissue in both CF and IPF lungs contained such areas, the nature of the inflammation was different. In accordance with the published literature, IPF lungs showed scattered areas with mild chronic inflammation with lymphocyte and mononuclear cell infiltrations, whereas CF lungs displayed a neutrophilic inflammation with signs of on going tissue destruction. The pro-inflammatory cytokine IL-6 was selected as an indicator of an "inflammatory" mast cell phenotype in this study. In CF lungs, both MC_T _and MC_TC _populations show elevated expression of IL-6 in all compartments investigated. IL-6 is a multifunctional regulator of immune and inflammatory processes [49] and is a potent chemo-attractant for neutrophils. In rat peritoneal mast cells it has been demonstrated that LPS, released from gram negative bacteria, induce IL-6 activation in absence of classical degranulation [50]. Therefore, during bacterial infections, a hallmark of CF, mast cells could via TLR receptors act as a source of IL-6 and other pro-inflammatory cytokines such as IL-1, IL-8, and GRO-α. The lower expression of IL-6 in IPF could indicate less active inflammation and lack of bacterial infections.

In the present study the density change of mast cell populations and altered MC_TC_/MC_T _balance was in the lung parenchyma restricted to areas affected by either fibrosis or cellular inflammation. Mast cells co-cultured with fibroblasts have been shown to induce collagen synthesis and stimulate fibroblast proliferation [2,4,35]. In relevance to the present study, co culturing of mast cells and fibroblasts induced a change in mast cell phenotypes, from MC_T _to MC_TC _[4]. This may indicate that the contact between mast cells and fibroblasts activate the cells involved, inducing processes leading to fibrosis and that MC_TC _plays an important role in this process. However, the elevated expression of mast cell IL-6 and TGF-β was present also in seemingly normal regions of the diseased lung. This observation suggests that, in the case of IPF, mast cells acquire a pro-fibrotic, high TGF-β expressing phenotype even prior to the formation of fibrotic regions. Similarly, in CF the elevated IL-6 expression in less affected regions suggests that mast cell activation in this disease may take place early in the pathogenesis. In support of this, based on findings of increased lung mast cell numbers already in CF foetuses, it has been speculated that mast cells may participate even in the initiation of the inflammation and lung histopathology in CF [51].

Medical treatment might have contributed to the change in MC_TC_/MC_T _proportion observed in the CF patients. Steroids have been demonstrated to reduce mast cell numbers and mainly affect the MC_T _population in various compartments of the lung [52]. All CF, but none of the IPF patients included in the study were treated with inhaled steroids and most of them with oral steroids. In CF patients this could be one explanation to the decrease in cell numbers in small airways and pulmonary vessels, accessible to steroids. However, the almost extinct mucosal mast cells in pulmonary vessels in CF may have other causes since a similarly robust reduction was seen in the untreated IPF patients. Regardless of treatment, increased mast cell numbers, particularly the MC_TC _population, in fibrotic and inflammatory region of the alveolar parenchyma was demonstrated in both CF and IPF patients. This might be explained by a particularly strong mast cell promoting milieu in the diseased parenchyma.

## Conclusions

In conclusion, the present study has demonstrated that increased numbers of altered MC_T _and MC_TC _mast cells infiltrate diseased regions of CF and IPF lungs. These results thus identify mast cell alterations as a significant and distinct part of the histopathology in CF and IPF. With mast cells being capable of participating in a both pro- and anti- inflammatory and fibrotic processes, explorations of the role of mast cells in CF and IPF lungs emerges as an important field of research for identifying novel strategies to combat these disorders.

## Competing interests

The authors declare that they have no competing interests.

## Authors' contributions

All authors have read and approved the final manuscript. CA contributed to the study design, data analysis, data collection, data interpretation, figures and writing of the manuscript. AA contributed to the study design, data interpretation, and writing of the manuscript. MM contributed to tissue collection and interpretation of the data. OH contributed to tissue collection, data interpretation and writing of the manuscript. AP contributed to the tissue collection, data interpretation and writing of the manuscript. LE contributed to the study design and data interpretation. LB supervised the study, contributed to data interpretation and writing of the manuscript. CL contributed to data interpretation and writing of the manuscript. MS contributed to the tissue collection, data interpretation and writing of the manuscript. GW contributed to the tissue collection, data interpretation and writing of the manuscript. JE supervised the study, contributed to study design, literature search, data interpretation and writing of the manuscript.
